# Bioinformatic-Experimental Screening Uncovers Multiple Targets for Increase of MHC-I Expression through Activating the Interferon Response in Breast Cancer

**DOI:** 10.3390/ijms251910546

**Published:** 2024-09-30

**Authors:** Xin Li, Zilun Ruan, Shuzhen Yang, Qing Yang, Jinpeng Li, Mingming Hu

**Affiliations:** Frontier Science Center for Immunology and Metabolism, Medical Research Institute, State Key Laboratory of Virology, Wuhan University, Wuhan 430072, China; lxin_z@whu.edu.cn (X.L.); zilunruan@whu.edu.cn (Z.R.); 2022206500036@whu.edu.cn (S.Y.); yqing2010@whu.edu.cn (Q.Y.)

**Keywords:** breast neoplasms, bioinformatics, tumor immunity, MHC-I molecules, interferon type I

## Abstract

Expression of major histocompatibility complex I (MHC-I) on tumor cells is extremely important for the antitumor immune response for its essential role in activating various immune cells, including tumor-specific CD8+ T cells. Cancers of lower MHC-I expression commonly exhibit less immune cell infiltration and worse prognosis in clinic. In this study, we conducted bioinformatic-experimental screening to identify potential gene targets to enhance MHC-I expression in breast cancer (BRCA). Through a combination of MHC-I scoring, gene expression correlation analysis, survival prognostication, and Cibersort tumor-infiltrated lymphocytes (TILs) scoring, we identify 144 genes negatively correlated with both MHC-I expression and TILs in breast cancer. Furthermore, we verified partially according to KEGG functional enrichment or gene-dependency analysis and figured out multiple genes, including *PIP5K1A*, *NCKAP1*, *CYFIP1*, *DIS3*, *TBP*, and *EXOC1*, as effective gene targets for increasing MHC-I expression in breast cancer. Mechanistically, knockout of each of these genes activated the intrinsic interferon response in breast cancer cells, which not only promoted MHC-I expression but also caused immunogenic cell death of breast cancer. Finally, the scRNA-seq confirmed the negative correlation of PIP5K1A et al. with TILs in breast cancer patients. Collectively, we identified multiple gene targets for an increase in MHC-I expression in breast cancer in this study.

## 1. Introduction

The low cure rate and high incidence of cancer result in millions of new cases and deaths annually. In most cases, the prognosis of cancer is poor, mainly due to immune evasion of cancer cells in patients [1]. Therefore, immunotherapy has emerged as a promising strategy for cancer treatment in addition to surgery, radiation therapy, and chemotherapy, particularly immune checkpoint blockade (ICB) therapy [1,2], though the effectiveness of cancer immunotherapy remains limited. According to the fundamental immunological principles, the key aspect of immunotherapy lies in recognizing antigens on tumor cells. Major histocompatibility complex-I (MHC-I) molecules bind to peptides expressed by cells and present antigen information on the cell surface, enabling immune cells, including CD8+ T cells, to identify abnormal protein-synthesizing pathological cells, such as cancer cells expressing mutant proteins [3,4]. However, the facts are that cancer cells usually employ certain mechanisms to evade elimination by CD8+ T cells so that they can survive and develop into tumor tissue [5]. One common mechanism for cancer cells to evade elimination is through loss of MHC-I antigen presentation. This not only impairs the intrinsic immune responses to transformed cancer cells but also hinders clinical immunotherapy [5,6,7,8,9]. In many cancers, the presence of tumor-infiltrating lymphocytes (TILs), which typically indicate the host immune response, is positively correlated with expression levels of MHC-I molecules on cancer cells [10,11]. MHC-I expression on tumor cells is regulated by both transcriptional and post-translational manners [12,13,14,15]. In most tumor cells, transcriptions of MHC-I family genes are predominately controlled by type I and II interferons, including interferon α/β and γ [12,16,17,18]. How to target this pathway to reactivate transcription of MHC-I family genes in MHC-I-low cancer types still needs further investigation.

Breast cancer is the most common malignant tumor in women. It is one of the cancers with the highest global incidence rate and ranks second in the mortality rate in women with cancer [19]. Breast cancer is divided into four molecular subtypes, including Luminal A type, Luminal B type, Her2 type, and Basal-like type, based on PAM50 gene expression profiling. Both Luminal A and Luminal B type breast cancers, accounting for about 70% of breast cancer patients, express estrogen receptors (ER) and are collectively referred to as estrogen receptor-positive (ER+) breast cancer [20]. Basal-like breast cancer (commonly triple-negative breast cancer, TNBC) accounts for 10–15% of all breast cancer cases, but it has the highest mortality rate due to the high aggressiveness and lack of effective targeted therapies [20]. Due to the large number of ER+ breast cancer patients, the total number of recurrences and deaths is far higher than that of triple-negative breast cancer patients [19].

Clinically, ER+ breast cancer has achieved significant clinical effects by using selective ER modulators or aromatase inhibitors to block the production of estrogen [21]. Additionally, combination therapy targeting CDK4/6 has been proven beneficial for ER+ breast cancer [22]. However, Luminal-type patients are not sensitive to chemotherapy, resulting in the need for alternative treatments once drug resistance occurs [22]. Currently, immunotherapy (such as immune checkpoint inhibitors) is mainly focused on patients of TNBC so far [23,24,25], since ER+ and Her2+ breast cancers are considered “cold tumors”, which are of low MHC-I expression, poor TIL infiltration, and insensitivity to chemotherapy [19,20,21,22]. Therefore, it is worth exploring strategies to enhance MHC-I expression in breast cancer, which will definitely benefit both the intrinsic anti-tumor immune response and immunotherapy efficacy during breast cancer treatment.

In this study, we conducted bioinformatics experimental screening to identify potential gene targets to enhance MHC-I expression in breast cancer. Through combination of MHC-I gene set variation analysis (GSVA) score, correlation analysis of gene expression, and survival prognostication, we identify 243 genes negatively correlated with MHC-I expression in breast cancer. We further found that expression levels of 144 out of 243 genes display a negative correlation with the tumor immune microenvironment indicated by the Cibersort score of TILs, including CD8+ T cells and NK cells. Next, we partially verified these candidates according to either KEGG analysis or gene-dependency analysis with Cancer Cell Line Encyclopedia (CCLE) BRCA cell lines, which leads to our identification of multiple genes, including *PIP5K1A*, *NCKAP1*, *CYFIP1*, *DIS3*, *TBP*, and *EXOC1*, as potent targets for enhancing MHC-I expression in breast cancer MCF7 cells. Mechanistically, bulk RNA-seq and hallmark gene set enrichment analysis (GSEA) indicated that knockout each of these genes in MCF7 cells activates the intrinsic interferon response in breast cancer cells, which is a predominant pathway to induce potent MHC-I expression. Furthermore, we found that knockout of each of these genes suppressed cell growth of breast cancer cells, which is probably due to interferon response-induced immunogenic cell death. Collectively, through bioinformatics-experimental screening, we identified multiple gene targets for enhancing MHC-I expression in breast cancer.

## 2. Results

### 2.1. Bioinformatics Analysis to Identify Potential Gene Targets for Enhancing MHC-I Expression in Breast Cancer

**Figure 1:**
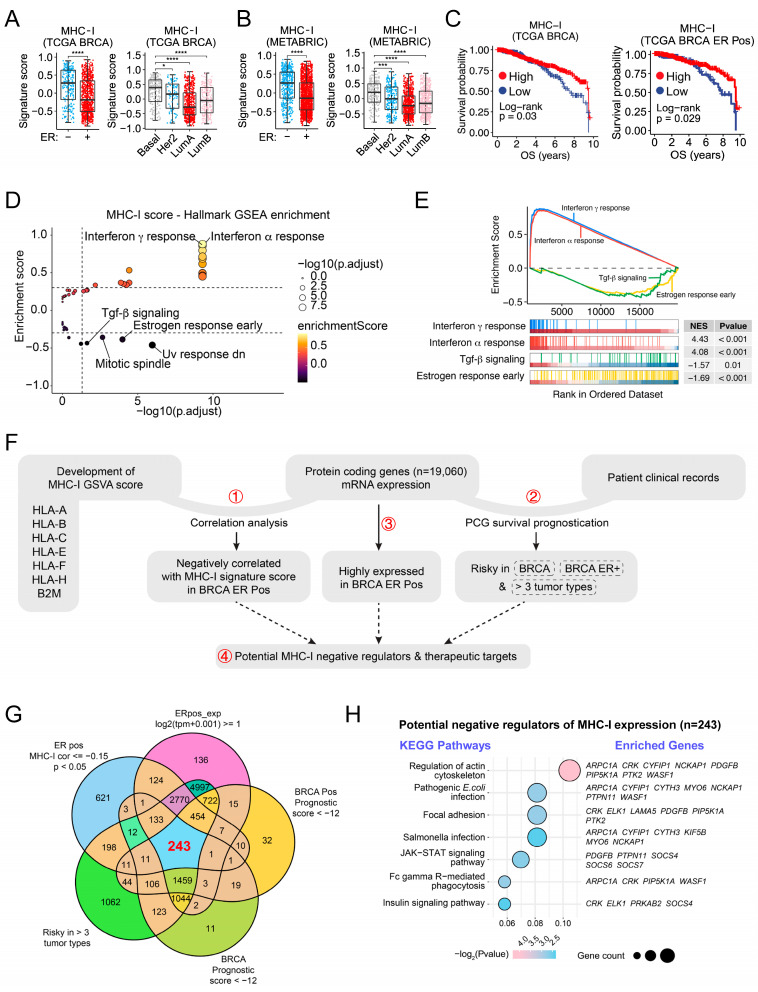
Bioinformatics analysis of gene targets for increase of MHC-I expression in breast cancer. (**A**,**B**) Boxplots display MHC-I signature scores for patients from the TCGA BRCA and METABRIC cohorts, categorized by ER status and breast cancer subtypes. In the TCGA BRCA cohort (**A**), scores are shown for ER-negative (*n* = 224) and ER-positive (*n* = 796) groups; subtypes include Basal (*n* = 191), Her2 (*n* = 81), LumA (*n* = 567), and LumB (*n* = 219). In the METABRIC cohort (**B**), ER statuses are negative (*n* = 609) and positive (*n* = 1817), with subtypes Basal (*n* = 209), Her2 (*n* = 224), LumA (*n* = 700), and LumB (*n* = 475). Each boxplot represents the median MHC-I signature score with whiskers indicating variability outside the upper and lower quartiles and individual data points plotted as dots. Statistical significance, determined using the Wilcoxon rank-sum test, is denoted by asterisks: **** for *p* < 0.0001, *** for *p* < 0.001, and * for *p* < 0.05. (**C**) Kaplan-Meier survival curves illustrating overall survival (OS) in both all (TCGA BRCA) and ER-positive (TCGA BRCA ER Pos) breast cancer patients with high (red) versus low (blue) MHC-I expression in the TCGA BRCA cohort. The *y*-axis indicates survival probability, and the *x*-axis represents OS in years. The *p*-values from the log-rank test are labeled. (**D**,**E**) GSEA based on MHC-I signature score in ER-positive breast cancer patients. Dot plot is shown in (**D**). The *x*-axis represents the negative logarithm of the adjusted *p*-value (−log10 (*p*.adjust)), while the *y*-axis denotes the enrichment score. The size of the bubbles corresponds to the adjusted *p*-value, and the color gradient indicates the enrichment score. Enrichment scores for the indicated pathways are shown in (**E**), with normalized enrichment scores (NES) and adjusted *p*-values. (**F**) A flow chart for identifying negative correlated factors of MHC-I expression by bioinformatics analysis. PCG, protein-coding genes. (**G**) Venn diagram illustrating the overlap of gene sets under different criteria in breast cancer. The diagram shows the intersection of five gene sets: (1) ER-positive (ER pos) breast cancer with MHC-I correlation less than or equal to −0.15 and *p*-value < 0.05 (blue), (2) genes with ER pos_exp (log2(tpm + 0.001)) ≥ 1 (pink), (3) BRCA-positive (BRCA Pos) with prognostic score less than −12 (yellow), (4) genes risky in more than three tumor types (green), and (5) BRCA with prognostic scores less than −12 (light green). The numbers within the diagram represent the count of genes falling into each category or intersection. The central intersection highlighted in red represents 243 genes common to all five criteria. (**H**) Bubble plot illustrating KEGG pathway enrichment analysis. Pathways are represented along the *y*-axis, with the *x*-axis displaying the gene ratio, defined as the proportion of genes within the pathway relative to the total number of input genes. Each bubble’s size reflects the gene count associated with the respective pathway, with larger bubbles indicating a higher number of genes. The color gradient of the bubbles corresponds to the statistical significance of the enrichment, represented by the −log2(*p*-value), where darker shades denote more significant *p*-values.

To achieve a comprehensive and objective evaluation of MHC-I expression in cancers, we developed a MHC-I signature score based on GSVA of eight MHC-I family genes, including *HLA-A*, *HLA-B*, *HLA-C*, *HLA-D*, *HLA-E*, *HLA-F*, *HLA-H*, and *B2M*. Pan-cancer analysis indicated that the MHC-I score was quite low in several cancer types, including breast cancer (Figure S1A), which is consistent with the previous observation by other groups [26,27,28,29,30,31]. Furthermore, we calculated the MHC-I signature score across different breast cancer types based on the Cancer Genome Atlas (TCGA) and the Molecular Taxonomy of Breast Cancer International Consortium (METABRIC) databases and found that the MHC-I score was much lower in ER+ (LumA or LumB) and Her2+ breast cancer types than that of Basal-like types (Figure 1A,B). Importantly, breast cancer patients with high MHC-I expression have a better prognosis than those with low MHC-I expression (Figure 1C), which encourages us to explore the potential strategies to enhance MHC-I expression in breast cancer. Hallmark GSEA indicated that MHC-I signature score in breast cancer samples from the TCGA database was markedly positively correlated with immune pathways, including interferon α/γ response (Figure 1D,E), while negatively correlated with procancerous pathways, such as estrogen response early and TGF-β signaling (Figure 1D,E), which indicates that targeting these pathways would be sufficient to enhance MHC-I expression in breast cancer.

To figure out potential gene targets for an increase in MHC-I expression in breast cancer, we designed a bioinformatics analysis process as shown in Figure 1F. We first figured out 4598 genes out of all protein-coding genes (*n* = 19,060) whose mRNA levels are negatively correlated with the MHC-I signature score (Step 1) (Figure S1B), and then figured out the risky genes in breast cancer according to survival prognostication (Step 2) (Figure S1C), followed by examination of whether they are highly expressed or not in breast cancer (Step 3). Genes fulfilling all three of the above conditions were selected as candidate genes (*n* = 243) (Figure 1G). Subcellular location annotations indicated that proteins coded by these 243 genes are distributed in different subcellular components, including the nucleus, cytoplasm, and various organelles (Figure S1D). KEGG analysis indicated that these genes could be predominately enriched in pathways related to immune response or infection (Figure 1H), and pathways of action cytoskeleton regulation and insulin signaling were also enriched (Figure 1H).

In conclusion, we performed bioinformatics analysis and identified multiple potential gene targets for enhancing MHC-I expression in breast cancer.

### 2.2. Bioinformatics Analysis to Identify Multiple Genes Negatively Correlated with Both MHC-I Expression and TILs in Breast Cancer

**Figure 2:**
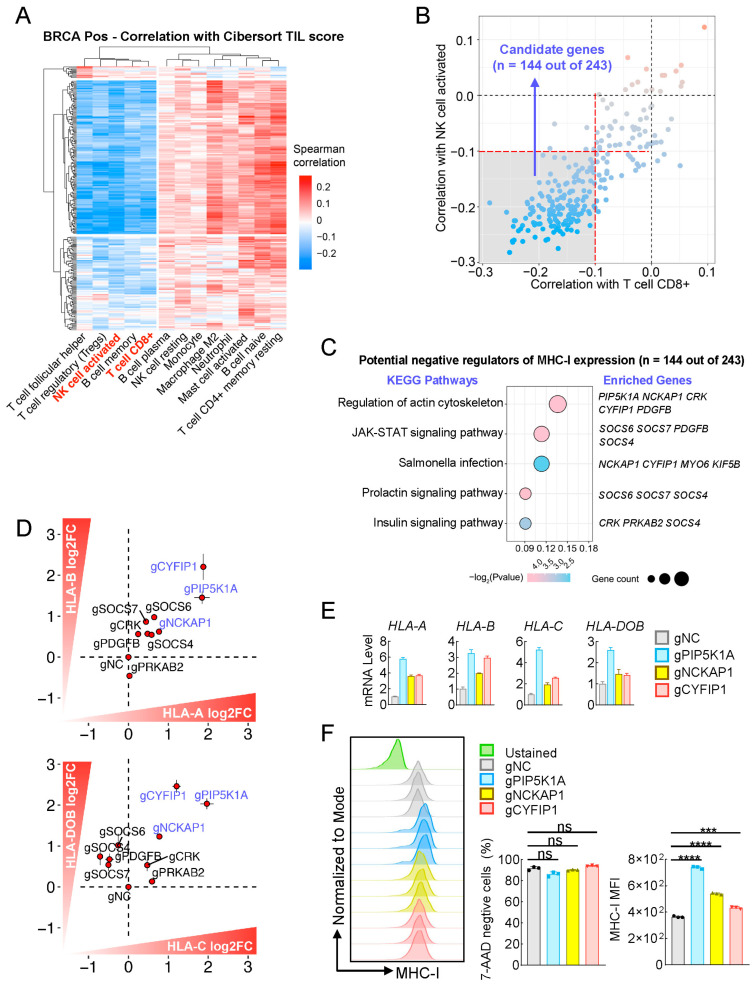
Partially verifying candidate genes for their role in the increase of MHC-I expression in breast cancer cells by gene knockout approach. (**A**) Clustered heatmap illustrating the Spearman correlations between 243 candidate genes (rows) and various TIL signatures (columns) in BRCA ER-positive samples. TIL signatures were computed using cell-type identification by estimating relative subsets of RNA transcripts (CIBERSORT), which estimates the relative proportions of immune cell types from gene expression data. The color scale ranges from blue (negative correlation) to red (positive correlation). Both genes and TIL types are hierarchically clustered, with dendrograms showing the relationship among them. This heatmap highlights key gene-TIL associations within the tumor microenvironment. (**B**) Scatter plot depicting the correlation between gene expression and two types of TIL scores, activated NK cells (*y*-axis) and CD8+ T cells (*x*-axis). Each point represents a gene, with the position determined by its correlation with activated NK cells or CD8+ T cells. The plot is divided into four quadrants by red and black dashed lines at correlation thresholds of −0.1 and 0.0. The shaded gray area highlights the lower left quadrant where both correlations are negative, indicating candidate genes (*n* = 144 out of 243) that are negatively correlated with both activated NK cells and CD8+ T cell scores (R < −0.1). (**C**) KEGG pathway enrichment analysis of genes identified from the analysis in (**B**). The *y*-axis lists the significantly enriched KEGG pathways, while the *x*-axis represents the gene ratio, calculated as the proportion of genes involved in each pathway relative to the total number of input genes. Each bubble’s size indicates the number of enriched genes in the respective pathway, and the color distinguishes different pathways. The right panel lists the specific genes associated with each pathway, highlighting their involvement in critical cellular processes. (**D**) Partially verifying the candidate genes for their role in increasing the transcription of MHC-I family genes in MCF7 cells. The indicated genes were knockout by the CRISPR-Cas9 method, followed by qPCR analysis of the mRNA levels of MHC-I family genes, including *HLA-A*, *HLA-B*, *HLA-C*, and *HLA-DOB*. Each red dot corresponds to a specific gene. (**E**) Effects of PIP5K1A-, NCKAP1-, or CYFIP1-deficiency on transcription of MHC-I family genes in MCF7 cells. The control (gNC) and PIP5K1A-, NCKAP1-, or CYFIP1-deficient (gPIP5K1A, gNCKAP1, gCYFIP1) MCF7 cells were harvested for qPCR analysis of the mRNA levels of the indicated genes. (**F**) Effects of PIP5K1A-, NCKAP1-, or CYFIP1-deficiency on MHC-I expression on the cell surface of MCF7 cells. The control (gNC) and PIP5K1A-, NCKAP1-, or CYFIP1-deficient (gPIP5K1A, gNCKAP1, gCYFIP1) MCF7 cells were harvested for flow cytometry analysis with PE-conjugated HLA-A2 antibody. The data shown are the mean ± SD (*n* = 3) from one representative experiment (**E**,**F**). ns, not significant, *** *p* < 0.001, **** *p* < 0.0001. Data are representative of three independent experiments.

To further screen out genes that are not correlated with tumor immune microenvironment signatures in clinical breast cancer, we conducted correlation analysis of expression of these 243 candidate genes with tumor immune microenvironment components by the Cibersort TILs score system. The results showed that expression of most of these genes is negatively correlated with scores of different TILs, including CD8+ T cells and NK cells activated (Figure 2A), two extremely important immune cells for antitumor immunity [32,33,34,35]. Next, we used these two types of immune cells for further calculation and found that 144 out of 243 candidate genes displayed markedly negative correlation (Spearman correlation coefficient < −0.1) with both CD8+ T cells and NK cells activated (Figure 2B). Furthermore, we analyzed the correlation between expression of these 144 genes with published immune-related signatures that also indicate the immune state in the tumors of patients, and we found that expression levels of all these genes were negatively correlated with signatures of MHC-I as well as IFNG_score (Figure S2A). Interestingly, positive correlations were observed between expression levels of most of these genes and the signature of PD-L1 (Figure S2A), indicating that targeting these genes in clinical cancer treatment might also suppress expression or effects of PD-L1, an important immune checkpoint expressed in cancer cells. Collectively, we identified multiple genes that negatively correlated with both MHC-I and TILs in breast cancer by bioinformatics analysis.

### 2.3. Partial Verification In Vitro to Identify Multiple Genes, Including PIP5K1A, NCKAP1, and CYFIP1, as Potent Targets for Enhancing MHC-I Expression in Breast Cancer

**Figure 3:**
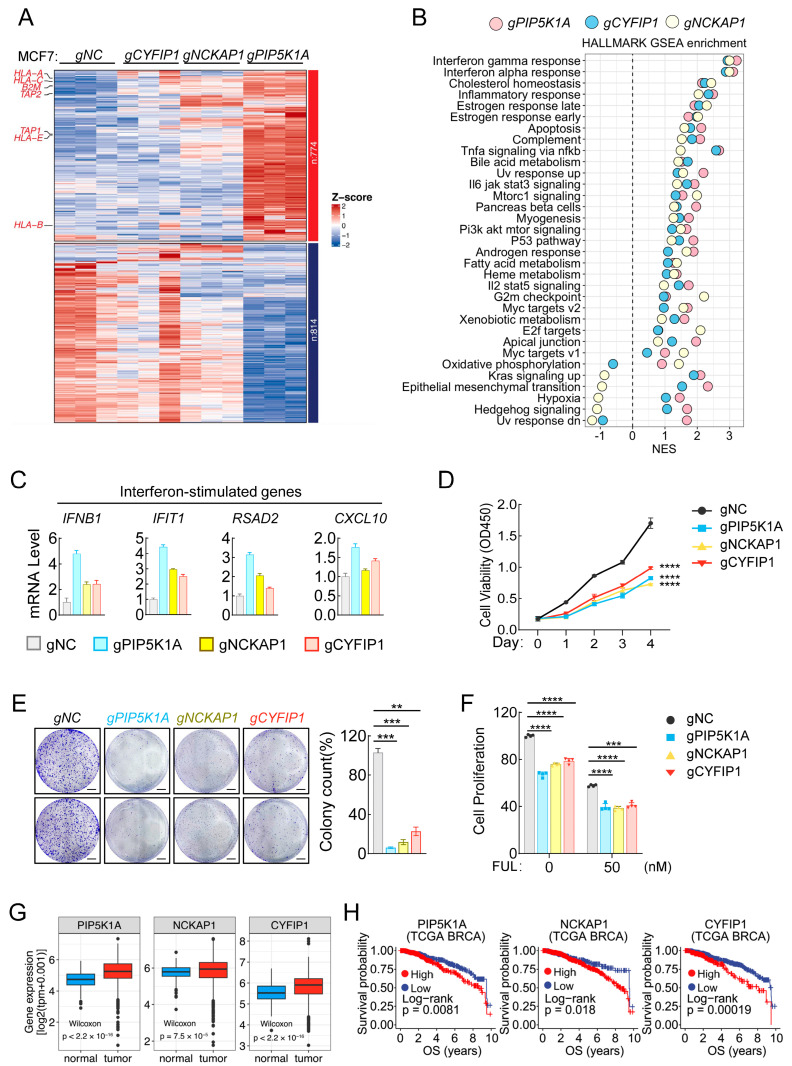
PIP5K1A-, NCKAP1-, or CYFIP1-deficiency activates the intrinsic interferon response and causes growth suppression of breast cancer cells. (**A**) Heatmap showing the expression profiles of significantly differentially expressed genes in PIP5K1A-, NCKAP1-, or CYFIP1-deficient MCF7 cells. Rows represent individual genes that were identified as significantly differentially expressed through differential expression analysis. Columns represent the different experimental conditions (gNC, gCYFIP1, gNCKAP1, gPIP5K1A). The color scale indicates the Z-score of gene expression, with red indicating upregulation and blue indicating downregulation. Key immune-related genes, including HLA-A, HLA-C, HLA-B, and TAP1/2, are highlighted on the left. The genes are clustered into two major groups based on their expression patterns, with the number of genes in each cluster shown on the right (*n* = 774 and *n* = 814). (**B**) Dot plot representing the results of hallmark GSEA following the deficiency of PIP5K1A (pink), CYFIP1 (blue), or NCKAP1 (yellow) in MCF7 cells. The *y*-axis lists various HALLMARK pathways, while the *x*-axis shows the normalized enrichment score (NES) for each pathway. Each dot represents the corresponding NES for a specific pathway. (**C**) Effects of PIP5K1A-, NCKAP1-, or CYFIP1-deficiency on transcription of interferon-stimulated genes. The control (gNC) and PIP5K1A-, NCKAP1-, or CYFIP1-deficient (gPIP5K1A, gNCKAP1, gCYFIP1) MCF7 cells were harvested for qPCR analysis of the mRNA of the indicated genes. (**D**) Effect of PIP5K1A-, NCKAP1-, or CYFIP1-deficiency on cell viability of MCF7 cells. The viability of the indicated cells was measured by CCK-8 assay. (**E**) Effect of PIP5K1A-, NCKAP1-, or CYFIP1-deficiency on colony formation of MCF7 cells. The clonogenic efficiency of the indicated cells was performed by a 2D colony formation assay. Shown are representative colony images (**left**) and quantification of colonies (**right**), scale bar: 5 mm. (**F**) Effect of PIP5K1A-, NCKAP1-, or CYFIP1-deficiency on MCF7 cell proliferation in the present or absent of fulvestrant treatment. The indicated cells were left un-treated or treated with fulvestrant (50 nM) for 36 h, followed by cell viability measurement by CCK-8 assay. (**G**) Boxplots showing the gene expression levels of PIP5K1A NCKAP1 and CYFIP1 in normal (blue, *n* = 292) and tumor (red, *n* = 1099) tissues derived from TCGA BRCA dataset. The *y*-axis represents gene expression levels measured in transcripts per million (TPM), log2-transformed [log2(tpm + 0.001)]. The horizontal lines within the boxes indicate the medians, while the boxes represent the interquartile ranges (IQR). Whiskers extend to data points within 1.5 times the IQR, with outliers depicted as individual points beyond the whiskers. *p*-values from the Wilcoxon rank-sum test are indicated for each comparison. (**H**) Kaplan-Meier survival curves illustrating overall survival (OS) probabilities for patients stratified by high (red) and low (blue) expression levels of PIP5K1A (High, *n* = 508; Low, *n* = 378), NCKAP1 (High, *n* = 358; Low, *n* = 517), and CYFIP1 (High, *n* = 616; Low, *n* = 254) in TCGA BRCA. The *y*-axis represents survival probability, and the *x*-axis indicates time in years. Log-rank *p*-values are labeled for each gene, indicating the statistical significance of the differences in survival outcomes between the high and low expression cohorts. The data shown are the mean ± SD (*n* = 3) from one representative experiment (**C**–**F**). ** *p* < 0.01, *** *p* < 0.001, **** *p* < 0.0001. Data are representative of three independent experiments.

Next, we determined to partially verify these 144 candidate genes in vitro. KEGG analysis indicated that these 144 genes could be enriched in pathways related to immune response, infection, action cytoskeleton, prolactin signaling, and insulin signaling (Figure 2C); thus, we chose these enriched genes (*n* = 10) to test their effect on transcriptions of MHC-I family genes. As shown in Figure 3C, knockout of the majority of these 10 genes by the CRISPR-Cas9 method increased transcriptions of MHC-I family genes, including *HLA-A*, *HLA-B*, *HLA-C*, and *HLA-DOB*. Among them, knockout of *PIP5K1A*, *NCKAP1*, and *CYFIP1* individually caused the greatest increase of gene transcription (Figure 2D,E and Figure S2B). To further confirm these results, we examined MHC-I expression on the cell surface of MCF7 with PE-conjugated anti-human HLA-A2 antibody by flow cytometry. We observed that knockout of these three genes individually indeed significantly enhanced MHC-I expression on MCF7 cell surface (Figure 2F).

In conclusion, we demonstrate that multiple genes, including *PIP5K1A*, *NCKAP1*, and *CYFIP1*, are effective targets for enhancing MHC-I expression in breast cancer cells.

### 2.4. Knockout of PIP5K1A, NCKAP1, and CYFIP1 Individually Activates Interferon Response and Inhibited Breast Cancer Cell Growth

To further figure out the underlying molecular mechanisms, we performed bulk RNA-seq with breast cancer MCF7 cell lines of control, as well as deficient of PIP5K1A, NCKAP1, or CYFIP1. The results showed that expression of many genes, including various MHC-I molecules, was upregulated in PIP5K1A-, NCKAP1-, or CYFIP1-deficient cells, especially in PIP5K1A-deficient cells (Figure 3A). Hallmark GSEA indicated that those upregulated genes were enriched in many different pathways related to immune response (interferon α/γ response, inflammatory response, Tnfα signaling, et al.), cell metabolism (cholesterol homeostasis, estrogen response, bile acid metabolism, et al.), cell apoptosis, and tumorigenesis, among which the interferon α/γ response exhibited the highest enrichment score (Figure 3B). As we noticed that the MHC-I signature score showed markedly positive correlation with the interferon a/g response in our previous bioinformatics analysis (Figure 1D,E), we wondered whether knockout of these three genes in breast cancer MCF7 cells activates the intrinsic interferon signaling and then promotes expression of interferon-stimulated genes (ISGs), which include various MHC-I family molecules [12,30,36,37,38]. The qPCR experiments showed that knockout of these three genes individually indeed induced expression of the interferon gene *IFNB1*, as well as downstream ISGs (such as *IFIT1*, *RSAD2*, and *CXCL10*) (Figure 3C). These results together suggest that knockout of *PIP5K1A*, *NCKAP1*, and *CYFIP1* individually activates the intrinsic interferon response to promote MHC-I expression in breast cancer cells.

It has been reported that the interferon response is an intrinsic antitumor pathway that not only induces expression of various immune molecules but also directly triggers immunogenic cell death in tumors [39,40,41]. Therefore, we further examined whether knockout of these three genes affects MCF7 cell growth in vitro. The results showed that knockout of each of these three genes markedly decreased cell viability of MCF7 in the CCK8 assays (Figure 3D) and inhibited colony formation of breast cancer MCF7 cells (Figure 3E). Furthermore, knockout of each of these three genes further inhibited cell proliferation to a higher degree in combination with fulvestrant, a classical drug in recurrent breast cancer treatment [21,42,43] (Figure 3F).

Finally, we evaluated the clinical significance of these three genes. Expression analysis showed that expression of all these three genes was increased in tumor tissues derived from the TCGA BRCA dataset (Figure 3G), and survival analysis indicated that high expression of these three genes displayed a poor prognosis in breast cancer patients (Figure 3H). All these results indicate that these three genes would be promising targets for breast cancer treatment in clinic.

### 2.5. Identification of Multiple Genes, Including DIS3, TBP, and EXOC1, as Potential Dual-Effectors That Regulate Both MHC-I Expression and Cell Survival in Breast Cancer

**Figure 4:**
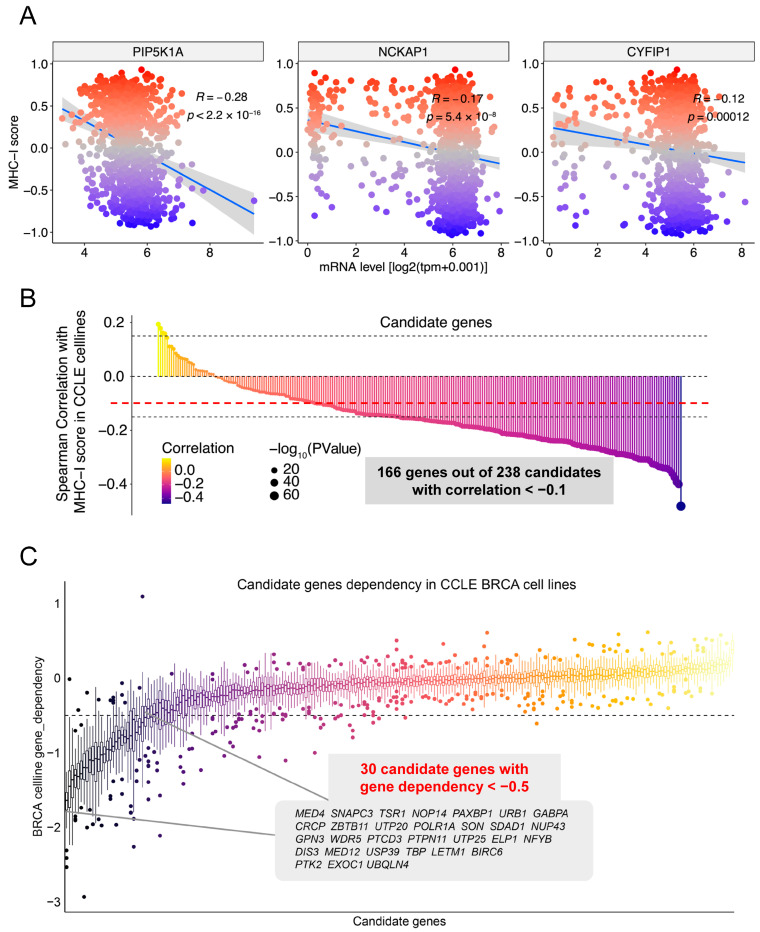
Bioinformatics analysis of data from the CCLE for the potential dual-effectors that regulate both MHC-I expression and cell survival in breast cancer. (**A**) Scatter plot of CYFIP1, NCKAP1, and PIP5K1A gene expression (log2-transformed) versus MHC-I signature scores across various breast cancer cell lines from the CCLE. Each dot represents a distinct cell line, color-coded by subtype. The plot includes a regression line (blue) with its 95% confidence interval (gray), illustrating a significant negative correlation between MHC-I scores and the indicated gene expression. (**B**) Lollipop plot displaying the Spearman correlation coefficients between the candidate genes and MHC-I scores across CCLE cell lines. Each line represents a gene, color-coded according to its correlation coefficient (cor) with MHC-I scores, ranging from yellow (positive correlation) to purple (negative correlation). The size of the dot at the end of each line corresponds to the statistical significance of the correlation, represented by −log10(*p*-value). Larger dots indicate higher statistical significance. (**C**) Boxplot analysis of gene dependency scores for the candidate genes across CCLE BRCA cell lines. The *y*-axis represents gene dependency, with lower scores indicating higher dependency. Each boxplot summarizes the distribution of dependency scores, with individual dots representing outliers. Genes with highly negative scores (left side, inset) are potential dual-effectors.

Induction of tumor cell killing by drug cytotoxicity and improvement of tumor immune microenvironment are two most important strategies for cancer treatment [33,42,44,45]. Therefore, we determined to identify potential dual-effectors that regulate both MHC-I expression and cell survival in breast cancer. We referred to the CCLE database for further bioinformatics analysis. Based on the CCLE dataset, we also observed the negative correlation of expression of *PIP5K1A*, *NCKAP1*, and *CYFIP1* with the MHC-I signature score (Figure 4A). Furthermore, we applied this analysis to all the 243 candidate genes and found that 166 out of these candidate genes were negatively correlated with the MHC-I signature score in CCLE breast cancer cell lines (Figure 4B). Finally, we analyzed the gene-dependency of these 166 candidate genes and figured out 30 genes of low gene-dependency (score < −0.5, which indicates an essential role for cell survival) (Figure 4C). Therefore, we identified 30 genes as potential dual-effectors that regulate both MHC-I expression and cell survival in breast cancer.

**Figure 5:**
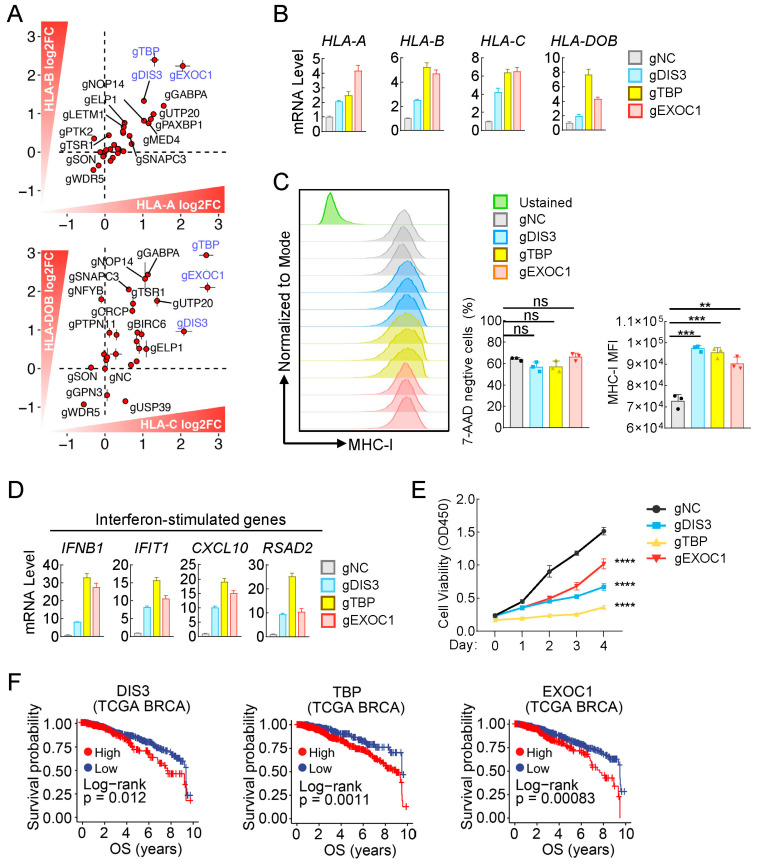
Identifying multiple genes as potential dual-effectors that regulate both MHC-I expression and cell survival in breast cancers. (**A**) Verifying the candidate genes for their role in increasing transcription of MHC-I family genes in MCF7 cells. The indicated genes were knockout by the CRISPR-Cas9 method, followed by qPCR analysis of the mRNA levels of MHC-I family genes, including *HLA-A*, *HLA-B*, *HLA-C*, and *HLA-DOB*. Each red dot corresponds to a specific gene. (**B**) Effects of DIS3-, TBP-, or EXOC1-deficiency on transcription of MHC-I family genes in MCF7 cells. The control (gNC) and DIS3-, TBP-, or EXOC1-deficient (gDIS3, gTBP, gEXOC1) MCF7 cells were harvested for qPCR analysis of the mRNA levels of the indicated genes. (**C**) Effects of DIS3-, TBP-, or EXOC1-deficiency on MHC-I expression on the cell surface of MCF7 cells. The control (gNC) and DIS3-, TBP-, or EXOC1-deficient (gDIS3, gTBP, gEXOC1) MCF7 cells were harvested for FACS analysis with PE-conjugated HLA-A2 antibody. (**D**) Effects of DIS3-, TBP-, or EXOC1-deficiency on transcription of interferon-stimulated genes. The control (gNC) and DIS3-, TBP-, or EXOC1-deficient (gDIS3, gTBP, gEXOC1) MCF7 cells were harvested for qPCR analysis of the mRNA of the indicated genes. (**E**) Effect of DIS3-, TBP-, or EXOC1-deficiency on cell viability of MCF7 cells. The viability of the indicated cells was measured by CCK-8 assay. (**F**) Kaplan-Meier survival curves illustrating overall survival (OS) probabilities for patients stratified by high (red) and low (blue) expression levels of DIS3 (High, *n* = 608; Low, *n* = 262), TBP (High, *n* = 309; Low, *n* = 562), and EXOC1 (High, *n* = 592; Low, *n* = 286) in TCGA BRCA. The *y*-axis represents survival probability, and the *x*-axis indicates time in years. Log-rank *p*-values are labeled for each gene, indicating the statistical significance of the differences in survival outcomes between the high and low expression cohorts. The data shown are the mean ± SD (*n* = 3) from one representative experiment (**B**–**E**). ns, not significant, ** *p* < 0.01, *** *p* < 0.001, **** *p* < 0.0001. Data are representative of three independent experiments.

To further verify these candidates, we knocked out these genes individually by the CRISPR-Cas9 method to evaluate their effects on MHC-I expression and cell survival in breast cancer MCF7 cells. Among all the 30 candidates, knockout of *DIS3*, *TBP*, or *EXOC1* individually enhanced transcription of MHC-I family genes to the highest degree (Figure 5A,B and Figure S3). Further flow cytometry experiments confirmed that knockout of *DIS3*, *TBP*, or *EXOC1* individually markedly increased MHC-I levels on the cell surface of breast cancer MCF7 cells (Figure 5C). Considering interferon response was one of the predominant pathways that regulate MHC-I expression in breast cancers according to our previous results (Figure 1D,E and Figure 3B), we wondered whether knockout of these three genes would also activate interferon response in breast cancer cells. The results showed that knockout of these three genes individually indeed induces expression of the interferon gene *IFNB1*, as well as downstream ISGs in MCF7 cells (Figure 5D). Finally, we evaluated whether these genes are essential for cell survival, and the CCK8 assays indicated that knockout of each of these three genes individually indeed decreased cell viability of MCF7 (Figure 5E). Additionally, survival analysis indicated that high expression of these three genes displayed a poor prognosis in breast cancer patients (Figure 5F), which indicates that these three genes would be promising targets for breast cancer treatment in clinic.

Conclusively, we identified multiple genes, including *DIS3*, *TBP*, and *EXOC1*, as potential dual-effectors that regulate both MHC-I expression and cell survival in breast cancer.

### 2.6. Correlation of Candidate Genes with MHC-I Expression and TME in Clinical Breast Cancer at Single-Cell Scale

**Figure 6:**
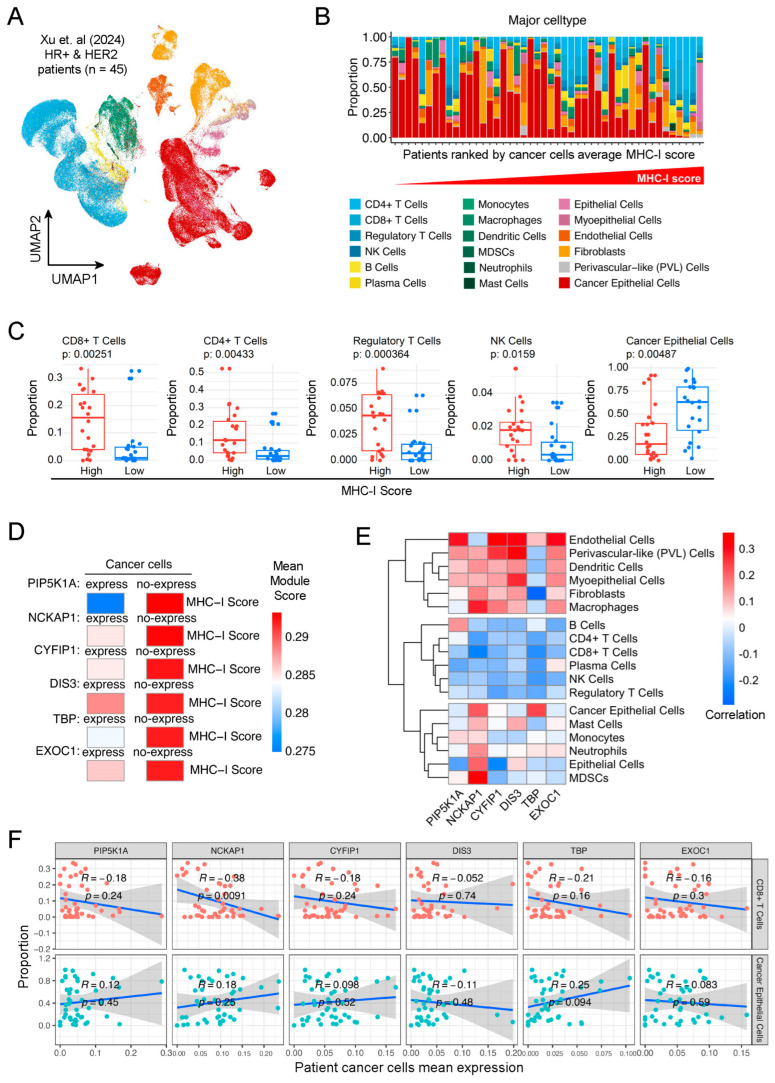
Correlation of candidate genes with MHC-I expression and TILs in clinical breast cancer at single-cell scale. (**A**) UMAP plot representing single-cell RNA sequencing data of the tumor microenvironment (TME) from HR+ and Her2+ breast cancer patients (*n* = 45). Each point corresponds to an individual cell, with colors indicating different major cell types within the TME. (**B**) Bar plot showing the proportions of major cell types within the TME across patients, arranged by their cancer cells’ average MHC-I score. Each bar represents a single patient, with colors depicting the various cell types present in the TME. (**C**) Boxplots displaying the proportions of CD8+ T cells, CD4+ T cells, regulatory T cells, NK cells, and cancer epithelial cells within the microenvironment of tumor samples categorized by high and low MHC-I expression. Statistical significance between the high (red) and low (blue) MHC-I groups was determined using the Wilcoxon rank-sum test, with *p*-values labeled for each cell type. The *y*-axis quantifies the proportion of each respective cell type. (**D**) Heatmap showing the mean MHC-I scores between cancer cells that express or not express specific genes (*PIP5K1A*, *NCKAP1*, *CYFIP1*, *DIS3*, *TBP*, *EXOC1*). The color gradient from blue to red represents the range of mean module scores, with blue indicating lower scores and red indicating higher scores. (**E**) Heatmap showing the correlation between the expression levels of selected genes (*PIP5K1A*, *NCKAP1*, *CYFIP1*, *DIS3*, *TBP*, *EXOC1*) in tumor cells and various cell populations within the tumor microenvironment. Rows represent different cell types, while columns correspond to the indicated genes. The color scale reflects the correlation coefficients, with red indicating positive correlations and blue indicating negative correlations. Hierarchical clustering was performed on both genes and cell types to identify patterns of association between gene expression and immune cell infiltration within the tumor microenvironment. (**F**) Correlation scatter plots showing the relationship between the average expression levels of selected genes in tumor cells and the proportions of CD8+ T cells and cancer epithelial cells within the tumor microenvironment. Each row corresponds to a specific cell type, while each column represents a gene of interest. The Spearman correlation coefficients (R) and *p*-values are indicated within each plot, with the blue line representing the fitted regression line and the shaded area indicating the 95% confidence interval.

To further elucidate the correlation of the above six genes with MHC-I expression and tumor microenvironment (TME) in clinical breast cancer, we referred to the published single-cell sequencing dataset from breast cancer patients [46] (Figure 6A). The proportions of major cell types within the TME across patients, arranged by their cancer cells’ average MHC-I score (Figure 6B). We divided these breast cancer samples into high and low MHC-I groups according to their MHC-I signature score shown in Figure 6B. The result showed that proportions of various tumor-infiltrated lymphocytes, including CD8+ T cells, CD4+ T cells, regulatory T cells, and NK cells, were much higher in the group with a high MHC-I score, while proportions of cancer epithelial cells were much lower in this group, which solidly supported the opinion that high MHC-I expression in tumors usually correlates with more TILs in TME.

To evaluate the correlation of MHC-I expression with the above six candidate genes, we calculated the mean MHC-I scores between cancer cells that express or do not express specific genes, including *PIP5K1A*, *NCKAP1*, *CYFIP1*, *DIS3*, *TBP*, and *EXOC1*. We found that MHC-I scores were higher in cancer cells without expression of *PIP5K1A*, *NCKAP1*, *CYFIP1*, *DIS3*, *TBP*, and *EXCO1* (Figure 6D). These results indicate that MHC-I expression was higher in cancer cells with low expression of these candidate genes, which was rightly consistent with the previous analysis from the TCGA dataset. Next, we analyzed the correlation of these six genes with TMEs in these clinical breast cancer samples. The results showed that expression of most of these six genes was negatively correlated with various TILs, including CD8+ T, CD4+ T, NK, and regulatory T cells in the TME of these breast tumors, while positively correlated with the proportions of cancer epithelial cells in these breast tumors (Figure 6E,F). Collectively, we demonstrated a negative correlation of the above six genes with MHC-I expression and TILs in the TME in clinical breast cancer at the single-cell scale.

### 2.7. MHC-I and Other Immune-Related Characteristic Analysis of the Six Candidate Genes across Different Cancer Types

Finally, we wondered whether these six candidate genes also regulate MHC-I expression and T lymphocyte infiltration in other cancer types in addition to breast cancer. Utilizing the methodologies described above, we conducted a comprehensive bioinformatic analysis of the prognostic characteristics of these genes within a pan-cancer context, as well as their correlations with different immune signatures, including MHC-I GSVA, CD8+ T cells, and NK cell scores (Figure S4A–D). As shown in Figure S4, NCKAP1 showed negative correlations with all these immune signatures and exhibited a poor prognosis across multiple cancers, including BRCA, cervical squamous cell carcinoma (CESC), head and neck squamous cell carcinoma (HNSC), liver hepatocellular carcinoma (LIHC), lung adenocarcinoma (LUAD), lung squamous cell carcinoma (LUSC), pancreatic adenocarcinoma (PAAD), skin cutaneous melanoma (SKCM), stomach adenocarcinoma (STAD), and uterine carcinosarcoma (UCS). PIP5K1A also exhibited negative correlations with these immune signatures and indicated a poor prognosis in BRCA, CESC, LIHC, PAAD, pheochromocytoma and paraganglioma (PCPG), prostate cancer (PRAD), rectum adenocarcinoma (READ), SKCM, testicular germ cell tumors (TGCT), and thyroid carcinoma (THCA). DIS3 demonstrated negative correlations with these immune signatures and exhibited a poor prognosis in BRCA, CESC, HNSC, LUSC, SKCM, and THCA. Similarly, CYFIP1 exhibited negative correlations with these immune signatures and showed a poor prognosis in tumors such as BRCA, LUSC, mesothelioma (MESO), sarcoma (SARC), and THCA. EXOC1 was linked to negative metrics in BRCA and SARC. Lastly, TBP displayed negative correlations with these immune signatures and exhibited a poor prognosis in BRCA and ovarian cancer (OV). Collectively, these findings suggest that these six candidate genes may also regulate MHC-I expression and T lymphocyte infiltration in other cancer types in addition to breast cancer.

## 3. Discussion

In many cancers, the presence of tumor-infiltrating lymphocytes (TILs), which typically indicate the host immune response, is positively correlated with expression levels of MHC-I molecules on cancer cells [10,11]. MHC-I expression is very low in breast cancer, especially in ER+ and Her2+ types, which disturbs the application of caner immunotherapy in these breast cancers. To figure out effective strategies for enhancement of MHC-I expression in breast cancer, we utilized a bioinformatics-experimental screening method to identify potential gene targets. This effort led to our identification of multiple genes, including *PIP5K1A* et al., as promising targets for an increase in MHC-I expression in breast cancer.

To obtain a reliable result of bioinformatics analysis, we took various factors into consideration, including whether highly expressed in breast cancer, whether negatively correlated with MHC-I expression, whether correlated with poor prognosis, as well as whether correlated with poor tumor immune microenvironment. During the verification, we utilized the gene knockout approach of CRISPR-Cas9 to examine their effect on transcriptions of MHC-I family genes, including *HLA-A, HLA-B, HLA-C*, and *HLA-DOB*, followed by analysis of MHC-I expression on tumor cell surfaces by the method of flow cytometry for further confirmation. After all these rigorous analyses and testing processes, we proved the effectiveness of most of the candidate gene targets. Therefore, our study not only uncovers multiple gene targets for enhancing MHC-I expression in breast cancer but also provides a model for identifying potential gene targets in other tumors with low MHC-I expression.

During the process of functional verification, we mainly focused on six candidate genes, knockout of which enhanced MHC-I expression to the highest degree. Through qPCR and flow cytometry, we confirmed that knockout of these six genes indeed significantly increased MHC-I expression in breast cancer MCF7 cells. Furthermore, we found that knockout of these genes could activate the intrinsic interferon response pathway in tumor cells. The interferon pathway predominately regulates transcription of various MHC-I family genes in most cells [12,16,17]. This suggests that proper activation of interferon signaling in breast cancer may be a key approach to improving MHC-I expression in breast cancer. Type I interferons are important active components of the immune system, and some types of interferons have been applied to the clinical treatment of certain cancers, but systemic inflammatory damage is usually an issue that cannot be ignored. Activating the intrinsic interferon response of tumor cells through suppression of certain genes would be a safer strategy, especially for tissue-specific gene targets. It has been reported that the interferon response is an intrinsic antitumor pathway that not only induces expression of various immune molecules, but also directly triggers immunogenic cell death in tumors [39,40,41]. We indeed observed cell growth arrest of MCF7 cells deficient of these six candidate genes in vitro. Of course, we noticed that deletion of *PIP5K1A*, *NCKAP1*, and *CYFIP1* genes also changes gene expression in certain metabolic pathways, such as cholesterol metabolism, which might also contribute to suppression of tumor cell growth induced by deficiency of these molecules. Through bioinformatics analysis, we found that *PIP5K1A*, *NCKAP1*, and *CYFIP1* are highly expressed in breast cancer tumors compared with normal tissues, and higher expression of these genes is correlated with a worse prognosis in breast cancer patients. This further highlights the clinical significance of these gene targets and their potentialities in clinical application.

How deletion of these six genes induces an intrinsic interferon response in breast cancer cells remains to be further studied. CYFIP1 is a component of the CYFIP1-EIF4E-FMR1 complex that plays a crucial role in translational repression by binding to the mRNA cap. DIS3 is a catalytic component of the RNA exosome complex, which is involved in the 3′ → 5′ exoribonuclease activity crucial for RNA processing and degradation. NCKAP1 is a part of the WAVE complex that regulates the formation of lamellipodia by controlling actin filament reorganization. PIP5K1A catalyzes the phosphorylation of phosphatidylinositol 4-phosphate to form phosphatidylinositol 4,5-bisphosphate, a lipid second messenger that regulates various cellular processes such as signal transduction, vesicle trafficking, and actin cytoskeleton dynamics. It also plays a role in phagocytosis, cell migration, and mRNA polyadenylation in nuclear speckles. TBP is a core component of the TFIID complex, which is essential for the initiation of RNA polymerase II-dependent transcription. EXOC1 is a component of the exocyst complex, which is crucial for the docking of exocytic vesicles with the plasma membrane. From the reported functions of these genes, we supposed that activation of the intrinsic interferon response may be related to disturbance of mRNA processing or actin cytoskeleton reorganization in breast cancer cells depleted of these genes.

During the study, we designed a new algorithm and used a bulk Cox regression analysis method across the entire genome to evaluate the relationship between the expression level of a specific gene in cancer patients and their survival period, thereby determining the prognostic role of the gene in different cancer types. The scoring system was able to quickly identify the role of genes as risk or protective factors in different situations, providing important references for biomedical research. The generated prognostic scores are intuitive and easy to understand, providing a data foundation for clustering analysis and pan-cancer research and providing direction for further exploring the prognostic performance of genes under different conditions. In practical applications, this method is easy to operate, and the results are intuitive, with strong compatibility that not only allows it to be integrated into existing analysis processes but also fully showcases the universality and specificity of gene function.

In this study, we utilized various bioinformatics tools for large-scale analysis. While bioinformatics tools offer significant advantages in large-scale data analysis, they also have limitations, such as data heterogeneity, potential biases introduced by algorithm choices, and dependence on result interpretation. Moreover, batch effects and differences in data processing between databases can affect the stability and reproducibility of the analysis results. To enhance the credibility of our findings, we implemented several measures, including cross-validation using multiple independent datasets, employing different analytical methods to confirm the consistency of results, and integrating biological experiments to validate key findings from our bioinformatics analysis. Future studies could improve the reliability and generalizability of findings by integrating more data sources and optimizing analytical algorithms. Furthermore, our analysis was primarily based on mRNA expression data, exploring the correlation between gene expression levels and immune-related networks. However, mRNA expression does not always accurately reflect actual protein expression levels and functional activity. Protein expression is influenced by various post-transcriptional modifications and translational regulatory mechanisms, and relying solely on mRNA data may overlook these critical regulatory aspects. Therefore, future research could incorporate proteomics data to provide a more comprehensive understanding of gene expression and functionality, thereby further validating and expanding our findings. Additionally, in analyzing single-cell datasets, we faced several inherent limitations. Although single-cell sequencing technology provides high-resolution insights into cellular heterogeneity and the complexity of the microenvironment, it also has challenges such as high data noise, complex cell type annotations, and high computational resource demands. Furthermore, obtaining and processing single-cell data requires strict quality control and standardized protocols; otherwise, technical biases and errors may be introduced, leading to the influence of the accuracy of downstream analyses. Despite employing advanced single-cell analysis tools and rigorous quality control standards in our study, we must acknowledge the limitations inherent to these technologies. Therefore, future research could combine various single-cell analysis methods and introduce additional biological validation techniques to enhance the accuracy and reliability of single-cell data analyses.

In this study, all the experimental verification was conducted in vitro. Further, the in vivo roles of these gene targets in improving MHC-I expression and TIL distribution in TME should be evaluated by using tumor-bearing mouse models. Additionally, it is also worth exploring whether these targets can synergize with ICB therapy to suppress tumor growth in vivo.

## 4. Materials and Methods

### 4.1. Reagents and Antibodies

Reagents including Fetal bovine serum (FBS) (Cellmax, Beijing, China), Penicillin and streptomycin (Citava, Boston, MA, USA), Dulbecco’s modified Eagle’s medium (FBS) (Gibco, New York, NY, USA), Puromycin (VWR, Radnor, PA, USA), Polybrene (Millipore, Burlington, MA, USA), TRIzol (Takara, Shiga, Japan), SYBR Green Supermix (Bio-Rad, Hercules, CA, USA), HiScript III Select RT SuperMix for qPCR (Vazyme, Nanjing, China), CCK-8 solution (Vazyme, Nanjing, China), and Fulvestrant (MCE, Shanghai, China), as well as antibodies including 7-aminoactinomycin D (7-AAD) (BD Biosciences, Franklin Lakes, NJ, USA), Anti-Human HLA-A2 PE (Biolegend, San Diego, CA, USA), and Mouse Isotype IgG2b PE (Biolegend, San Diego, CA, USA), were purchased from the indicated manufacturers.

### 4.2. Cell Lines and Cell Culture

The HEK-293T cells were purchased from the American Type Culture Collection (ATCC). The MCF7 cells were kindly provided by Dr. Cheguo Cai (Wuhan University, Wuhan, China). These cell lines were cultured in DMEM (GIBCO) medium supplemented with 10% FBS and 1% penicillin-streptomycin (Citava) under an atmosphere of 5% CO_2_ at 37 °C. All cells were negative for mycoplasma.

### 4.3. Plasmid Construction and Cell Transfection

CRISPR-Cas9 gRNA plasmids for the indicated 40 candidate genes were constructed by standard molecular biology technique. HEK-293T cells were seeded into plates for 20 h, and then cells were transfected by standard calcium phosphate precipitation method at a density of 40–50%.

### 4.4. Gene Knockout by CRISPR-Cas9

Gene knockout in MCF7 cells was achieved using a CRISPR-Cas9 system. Briefly, double-stranded oligonucleotides corresponding to the target sequences were cloned into the lenti-CRISPR-V2 vector, and then these lentiviral plasmids were co-transfected with two packaging plasmids into HEK-293T cells. Two days after transfection, the lentiviral supernatants were harvested, ultra-filtrated (0.45 mm filter, Millipore, Burlington, MA, USA), and then infected MCF-7 cells in the presence of polybrene (8 mg/mL). The infected cells were selected with puromycin (2 mg/mL) for at least 6 days to establish stable cell lines. The sequences targeted for the indicated genes were listed in Supplementary Table S1.

### 4.5. RT-qPCR

Total RNAs were extracted from cells with TRIzol (Takara) according to the manufacturer’s manual. The reverse-transcribed products were obtained for qPCR analysis to measure mRNA levels of the indicated genes. Data shown were the relative abundance of the indicated mRNA normalized to that of GAPDH. The qPCR data were collected with Bio-Rad CFX96 (Version 3.1) and analyzed with Bio-Rad CFX Maestro (Version 2.2). The sequences used for the RT-qPCR primers are listed in Supplementary Table S2.

### 4.6. Flow Cytometry

For analysis of MHC-I level on cell surface of the control, as well as PIP5K1A-, NCKAP1-, CYFIP1-, DIS3-, TBP-, or EXOC1-deficiency MCF7 cells, cells (5 × 10^5^) were seeded into 12-well plates (80–90% density), cells were subjected to staining with MHC-I (anti-human HLA-A2 antibody) and the isotype control (Mouse IgG2b, κ Isotype Ctrl Antibody) that are conjugated with PE. Stains were conducted at 4 °C for 30 min in the dark. For the dead cell exclusion analysis, cells were incubated for 10 min with 7-AAD before analysis. Flow cytometry acquisition was performed on a Fortessa™ X-20 flow cytometer (BD Biosciences, Franklin Lakes, NJ, USA), and data were analyzed with FlowJo software version 10.8.1.

### 4.7. Colony-Formation Assay

Cells were seeded in triplicate in 6-well plates at a density of 1000 cells per well, and then cultures were continued for 10–14 days, followed by fixing with 4% paraformaldehyde and staining with 0.2% crystal violet solution before subjected to photography. Image J software (v1.54g) was used for analysis.

### 4.8. Cell Counting Kit 8 (CCK8) Assay

Cell viability of MCF7 cells was assessed using the CCK-8 solution (Vazyme). Cells were seeded into 96-well plates in sextuplicate at a density of 5 × 10^4^ cells per well and cultured for the indicated times before 10 µL CCK8 solution was added into each well. The absorbance at 450 nm was then measured after incubation at 37 °C for 0.5–1 h.

### 4.9. RNA Extraction, Library Preparation, and Sequencing

RNA-seq experiments and high-throughput sequencing were conducted by Seqhealth Technology Co., LTD. (Wuhan, China). Approximately 1 × 10^7^ cells were used for RNA extraction, and DNA digestion was carried out by DNaseI after RNA extraction. The quality of RNA was determined by examining A260/A280 and 1.5% agarose gel electrophoresis. Qualified RNAs were finally quantified by Qubit3.0 with QubitTM RNA Broad Range Assay kit (Life Technologies, Carlsbad, CA, USA). Total RNAs (2 mg) were used for stranded RNA sequencing library preparation. PCR products corresponding to 200–500 bps were enriched, quantified, and finally sequenced on DNBSEQ-T7 sequencer (MGI Tech Co., Ltd., Shenzhen, China) with PE150 model.

### 4.10. RNA-Seq Analysis

In the genome-wide differential gene expression analysis, FASTQ files were first trimmed using Trim_galore (v0.6.9) to remove sequencing adapters and low-quality reads (Q < 15). Trimmed sequencing reads were aligned to the human Hg38 reference genome (GENCODE, GRCh37.p13) using STAR (v2.7.10a). SAM files were subsequently converted to BAM files, sorted, and indexed using samtools (v1.15). BAM files were used to generate bigwig files using bamCoverage (part of the Deeptools package; v3.5.1). Read counting across genomic features was performed using featureCounts (part of the subread package; v2.0.1).

Differential gene expression analysis was performed using DESeq2 (v1.34.0). Raw count data were normalized, and genes with low counts were filtered out to reduce noise. Differentially expressed genes (DEGs) were identified based on a false discovery rate (FDR) adjusted *p*-value < 0.05 and a log2 fold change (log2FC) threshold of ±1. Hierarchical clustering of DEGs was conducted using the pheatmap package (v1.0.12), employing Euclidean distance and complete linkage as the distance metric and clustering method, respectively. Clustering results were visualized in heatmaps, highlighting patterns of gene expression across different conditions.

KEGG pathway enrichment analysis was conducted using the clusterProfiler package (v4.0.5). Differentially expressed genes were mapped to KEGG pathways, and enrichment analysis was performed to identify significantly enriched pathways. Hallmark GSEA was performed using the MSigDB HALLMARK gene sets. The GSEA algorithm was applied using the fgsea package (v1.18.0), with gene sets ranked by their log2FC values from the differential expression analysis. Enriched gene sets were identified based on an FDR-adjusted *p*-value < 0.05. The normalized enrichment score (NES) was calculated for each gene set, and results were visualized in enrichment plots, highlighting key pathways and processes implicated in the analysis.

### 4.11. Clinical Data Analysis

To evaluate survival outcomes in breast cancer, we utilized primary solid tumor samples from the breast cancer cohort provided by TCGA or METABRIC. The access date for the TCGA database was 15 March 2024, with data obtained from UCSC Xena, using the integrated version of 2016-12-29. The access date for the METABRIC database was 25 March 2024, and the data were downloaded from cBioPortal, based on the version referenced in the publication “The somatic mutation profiles of 2433 breast cancers refine their genomic and transcriptomic landscapes”. The expression data were normalized using the TCGAbiolinks package (v2.22.1) and subsequently transformed with DESeq2 (v1.34.0) under default settings. ensured that the expression data were suitable for downstream analyses.

To assess pathway activity within the tumor samples, GSVA was performed using the GSVA package (v1.40.1). Firstly, the normalized gene expression data were input into the GSVA function, which computes a non-parametric, unsupervised estimation of variation in gene set enrichment across the samples. The analysis was conducted using a predefined list of gene sets, specifically focusing on immune-related pathways. The gene sets were mapped to the expression data, and for each sample, the GSVA algorithm calculated enrichment scores for each pathway by integrating expression levels of the corresponding genes; the resulting enrichment scores were then used for downstream analysis.

The quantification of TILs was performed using CIBERSORT (v1.06), a deconvolution algorithm that estimates the relative abundance of immune cell types within a mixed cell population based on gene expression data. Firstly, the normalized gene expression data were uploaded to the CIBERSORT web portal, where the LM22 signature matrix, which defines 22 immune cell subtypes, was applied. The algorithm then used a support vector regression model to deconvolute the expression profiles, providing an estimated proportion of each immune cell type within the tumor samples. The output included detailed percentages of various immune cell populations, allowing for an in-depth characterization of the immune microenvironment within each tumor sample.

Among the 1098 patients in the dataset, we specifically focused on the ER+ patients and those identified as the LumA molecular subtype through the PAM50 classification algorithm. Survival outcomes were analyzed using Kaplan-Meier survival curves, which were generated using the survival package (v3.2-13) and statistically evaluated with log-rank tests to determine the significance of differences between groups.

To explore the relationships between gene expression levels and other factors, including GSVA scores, TIL quantifications, and immune-related signatures, the Spearman correlation analysis was conducted. This analysis was performed using the GGPUBR package (v0.4.0) to provide a robust and comprehensive assessment of how these variables interrelate. The correlations were visualized with scatter plots, and correlation coefficients were calculated to identify significant associations.

The overall procedure for prognosis scoring is outlined as follows: Curated clinical data, including subtype and stratification information, was linked to integrated survival and expression data. Unlike traditional single Cox regression analysis methods, this approach utilizes a multi-section, multi-time-point perspective. It processes multiple cancer types in batches and conducts stratified analyses across various survival indicators, such as overall survival, disease-specific survival, progression-free survival, and time to event. This comprehensive approach systematically addresses the complex relationships between different cancer types and survival periods. By performing Cox proportional hazards analysis at multiple time points, the method mitigates the risk of bias introduced by relying on a single cut-off time, allowing for a more accurate assessment of the dynamic relationship between gene expression and patient survival, thereby improving the robustness and reliability of the analysis.

### 4.12. Cancer Cell Line Encyclopedia (CCLE) Dataset Analysis

To explore cancer epithelial cell heterogeneity and gene-cell dependency, we obtained bulk RNA-seq data of human breast cancer cell lines from the Broad Cancer Cell Line Encyclopedia (CCLE) DepMap portal (version 24Q1). The access date for the CCLE database is also 15 March 2024, with data from version 24Q1. Bulk RNA-seq data from CCLE containing TPM values of protein-coding genes were inferred using the RSEM tool and loaded into Seurat (v.5.1.0) and log-normalized. For each cell line, GE expression was calculated by the UCell (v1.99.1) score of the signature. UCell (v1.99.1) scores were Z scores normalized across all breast cancer cell lines.

To assess the immunogenic potential of each cell line, we applied the MHC-I signature to the normalized expression matrices for each cell line, performing a GSVA to calculate the corresponding score. The GSVA score indicated a quantitative measure of the MHC-I activity in each cell line. To evaluate the importance of specific molecules, we cross-referenced the dependency data with the GSVA scores. Spearman correlation was used to assess the relationship between candidate gene expression and MHC-I scores in breast cancer cell lines.

### 4.13. Single-Cell Data Analysis

In this study, we leveraged the integrated single-cell RNA sequencing (scRNA-seq) dataset provided by the publication “A comprehensive single-cell breast tumor atlas defines epithelial and immune heterogeneity and interactions predicting anti-PD-1 therapy response”. The dataset includes 236,363 single cells isolated from 119 biopsy samples across eight distinct datasets, offering a comprehensive view of the breast tumor microenvironment (TME).

Our analysis focused on low MHC-I-expressed cancer types, including HR+ and Her2+ breast cancer patients (*n* = 45), using the provided cell type annotations to investigate the heterogeneity within both immune and epithelial cell populations. Single-cell RNA-seq data were processed using the standard Seurat workflow, which included normalization with the LogNormalize method followed by scaling and principal component analysis (PCA) for dimensionality reduction. Clustering was performed using the default settings, and cell populations were annotated based on marker gene expression as provided in the original study.

To validate the potential regulatory roles of candidate molecules identified in prior analysis, the average expression levels of these genes were computed within the tumor cell populations of patients. Immune deconvolution was subsequently performed to assess the proportion of various immune cell types, including tumor-infiltrating lymphocytes (TILs). To ensure the robustness of the findings, samples with below 10% or above 90% tumor cell proportion were excluded. Finally, the Spearman correlation coefficients were calculated to explore the relationship between gene expression levels and TIL infiltration scores.

### 4.14. Statistics Analysis

All experiments were repeated at least three times with similar results, and the data represented in bar graphs are shown as mean ± SD (*n* = 3) from one representative experiment. Statistics analysis was performed, and graphs were prepared with GraphPad Prism 8. Statistical differences were analyzed using Student’s unpaired *t*-test or one-way ANOVA. The number of asterisks represents the degree of significance with respect to *p* values. *p* < 0.05 (*), *p* < 0.01 (**), *p* < 0.001 (***), *p* < 0.0001 (****), ns, not significant scores.

## Data Availability

All data are provided in the article and its Supplementary File, or by the corresponding author upon reasonable request.

## Supplementary Materials

The following supporting information can be downloaded at: https://www.mdpi.com/article/10.3390/ijms251910546/s1.
