# Supplementary material for: Bioinformatic-Experimental Screening Uncovers Multiple Targets for Increase of MHC-I Expression through Activating the Interferon Response in Breast Cancer
Source: Int J Mol Sci. 2024 Sep 30;25(19):10546. doi: 10.3390/ijms251910546 (PMC11476581; doi:10.3390/ijms251910546)
Supplement: Supplementary file 1 [file ijms-25-10546-s001.zip › ijms-3227827-supplementary Figures.pdf]

International Journal of
Molecular Sciences

MDPI

Supplementary Materials for

Bioinformatic-experimental screening uncovers multiple targets for increase of MHC-I expression through activating the interferon response in breast cancer

Xin Li, Zilun Ruan, Shuzhen Yang, Qing Yang, Jipeng Li, and

Mingming Hu

This PDF file includes:

Figures S1 to S4

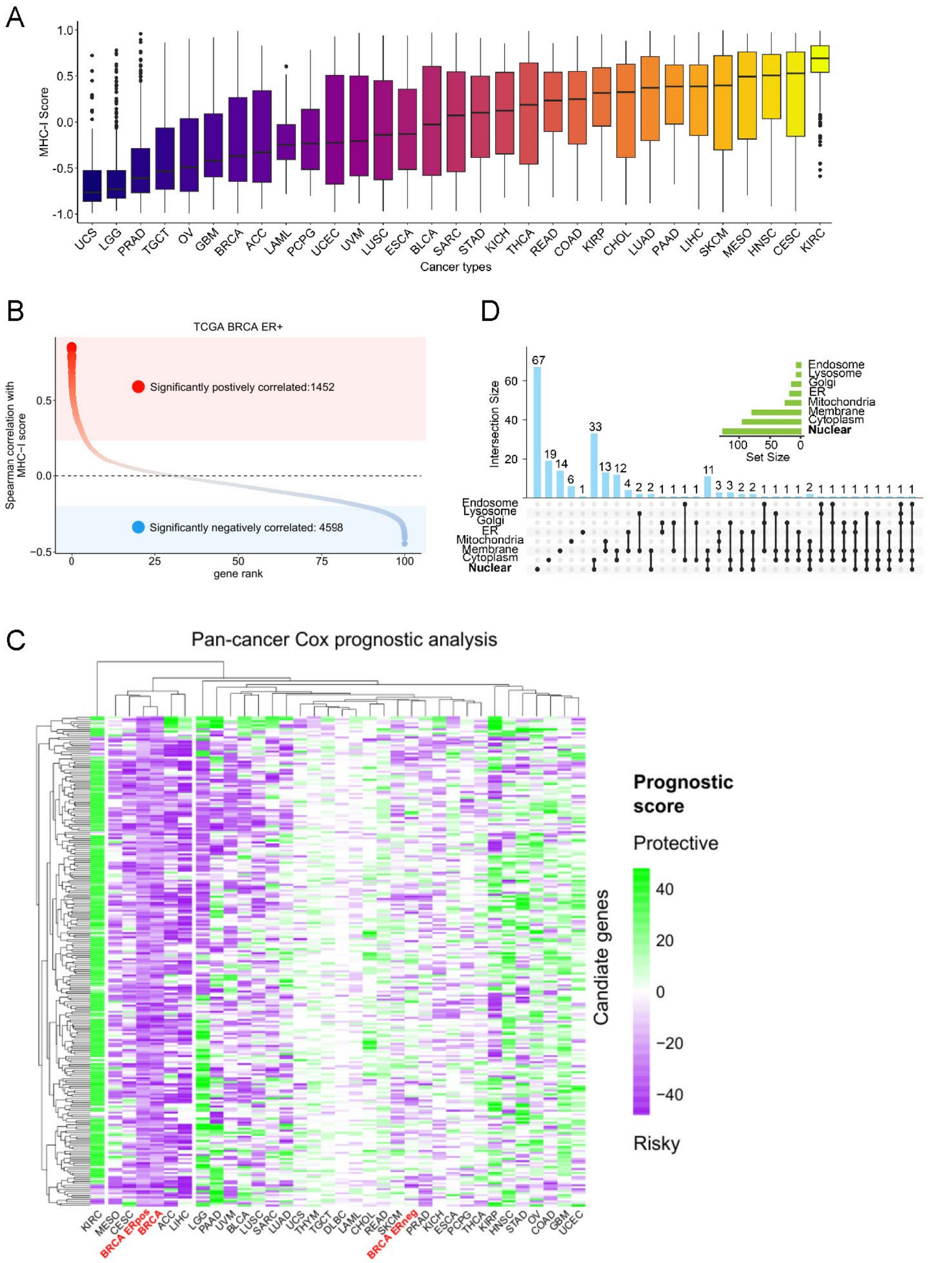

Figure S1. Bioinformatics analysis of gene targets for increase of MHC-I expression in breast cancer. **(A)** Boxplot illustrating the distribution of MHC-I scores across various cancer types based on data

from TCGA. Each box represents the interquartile range (IQR) of MHC-I scores within a specific cancer type, with the median value indicated by the horizontal line inside the box. Whiskers extend to data points within 1.5 times the IQR, and outliers are represented by individual points beyond the whiskers. **(B)** Scatter plot illustrating the Spearman correlation between gene expression and MHC-I scores in TCGA BRCA ER+ samples. The y-axis represents the Spearman correlation coefficient, while the x-axis shows the rank of genes based on their correlation with the MHC-I score. Genes significantly positively correlated with MHC-I scores are highlighted in red, with 1,452 genes in this category. Genes significantly negatively correlated are highlighted in blue, with 4,598 genes. **(C)** Heatmap illustrating the prognostic impact of candidate genes across multiple cancer types, as determined by the Cox proportional hazards model. The system utilizes a Cox proportional hazards model to assess the impact of specific genes on patient prognosis. Each gene is assigned a score, ranging from -48 to 48. Rows correspond to individual candidate genes, while columns represent different cancer types. The prognostic score is color-coded, with green indicating protective genes (positive scores) and purple indicating risky genes (negative scores) for patient outcomes. **(D)** UpSet plot showing the intersections and sizes of candidate protein sets localized to various cellular compartments. The top bars represent the size of each intersection, with the height indicating the number of proteins shared among the intersected compartments. The inset bar plot on the right displays the total set size for each cellular compartment.

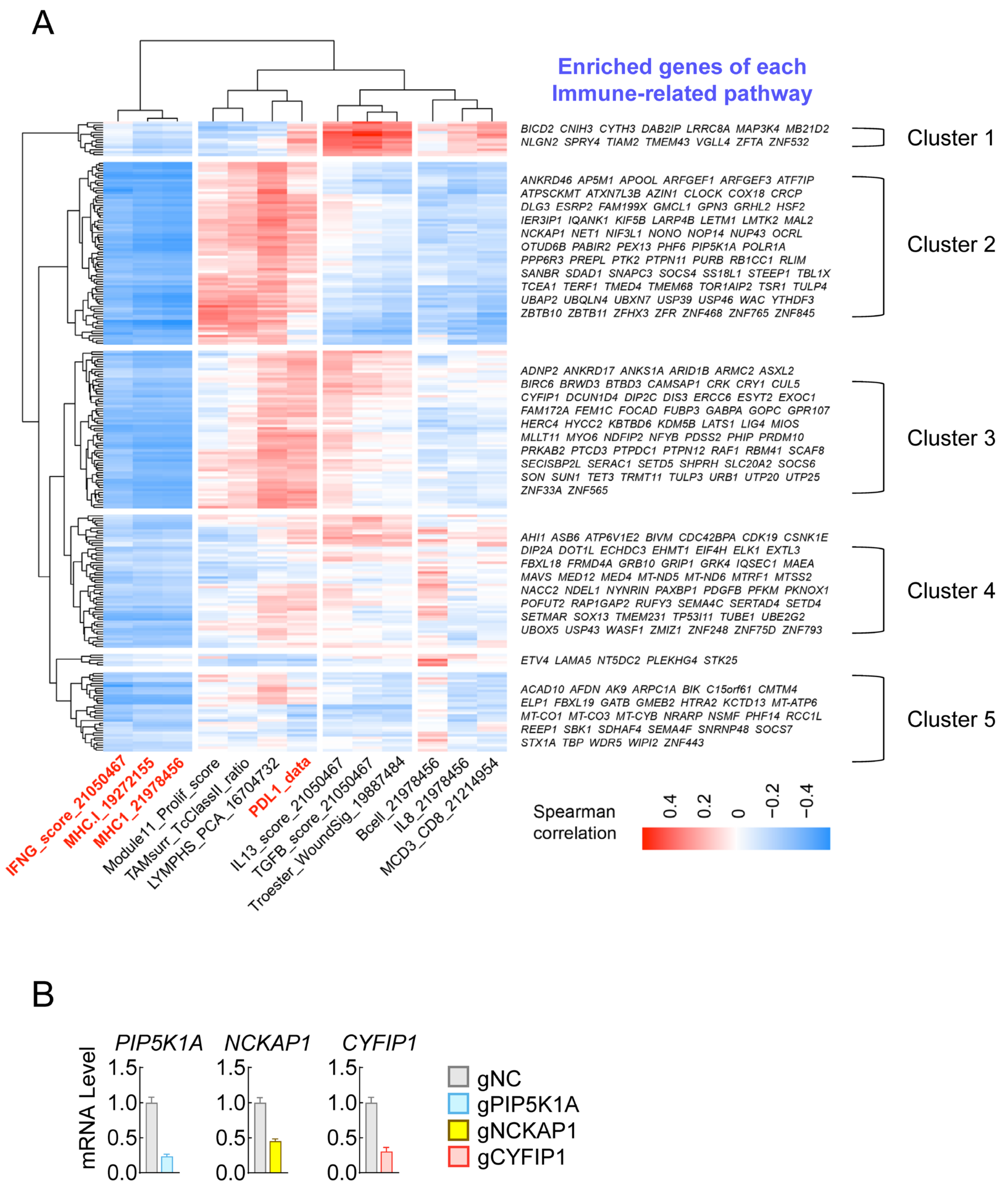

Figure S2. Correlation analysis of candidate genes with immune-related signatures. **(A)** Clustered heatmap showing the Spearman correlations between enriched genes from various immune-related pathways (rows) and published immune signatures (columns). The heatmap is organized into five clusters based on hierarchical clustering, as indicated by the dendrogram on the left. The columns represent different immune signatures, including MHC-I, IFNG, TGF-β, and others. The color scale represents the correlation coefficients, ranging from blue (negative correlation) to red (positive correlation). Each cluster groups genes with similar correlation patterns across the immune signatures, revealing distinct modules of gene-signature associations. **(B)** Examination of their respective mRNA levels in PIP5K1A-, NCKAP1- or CYFIP1-deficiency MCF7 cells. The indicated cells were harvested for qPCR analysis of the mRNA levels of the indicated genes.

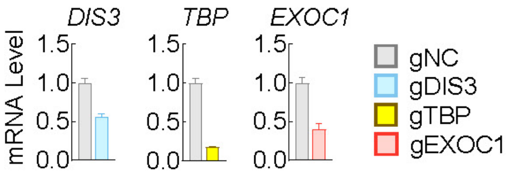

Figure S3. Examination of their respective mRNA levels in DIS3-, TBP- or EXOC1-deficient MCF7 cells. The indicated cells were harvested for qPCR analysis of the mRNA levels of the indicated genes.

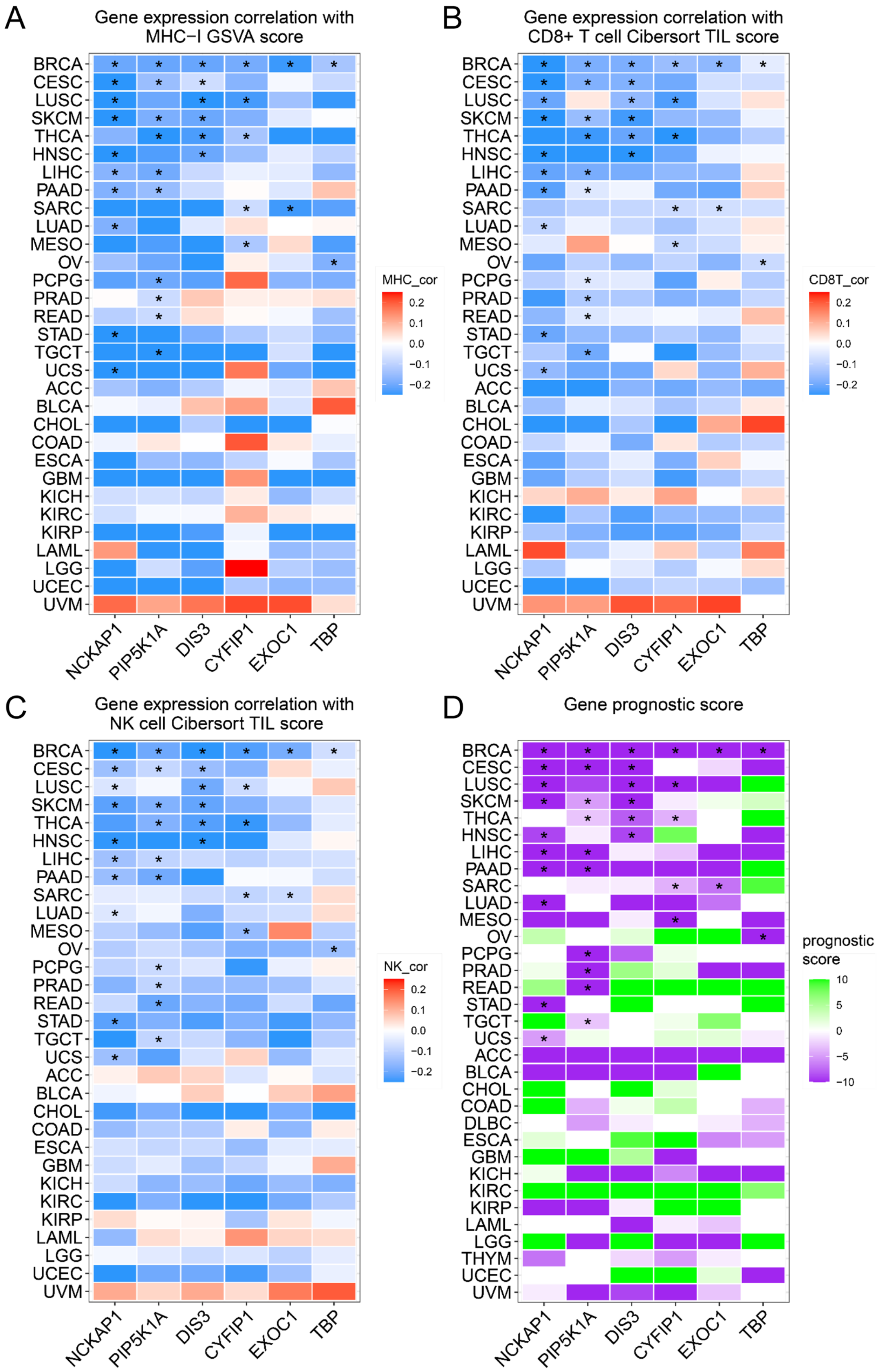

Figure S4. MHC-I- and other immune-related characteristic analysis of the six candidate genes across different cancer types. **(A)** Heatmap illustrating the Spearman correlation between the six genes and MHC-I GSVA scores across diverse cancers. Color scale ranges from blue (negative

correlation) to red (positive correlation). Columns correspond to individual candidate genes, while rows represent different cancer types. (B) Heatmap illustrating the Spearman correlation between the expression of six genes and CD8+ T cell infiltration across various cancer types, assessed using CIBERSORT TIL scores. Color scale ranges from blue (negative correlation) to red (positive correlation). (C) Heatmap illustrating the Spearman correlation between the six genes and NK cell infiltration in multiple cancers, derived from CIBERSORT TIL scores. Color scale indicates correlation strength from blue (negative) to red (positive). (D) Heatmap illustrates the prognostic impact of six candidate genes across multiple cancer types, as determined by the Cox proportional hazards model. The system utilizes a Cox proportional hazards model to assess the impact of specific genes on patient prognosis. Each gene is assigned a score, ranging from -48 to 48. The prognostic score is color-coded, with green indicating protective genes (positive scores) and purple indicating risky genes (negative scores) for patient outcomes. Asterisks (*) in the heatmap indicate cancer-gene combinations that pass all four tests. Specifically, these combinations meet the following criteria: a prognostic score less than or equal to -3, along with correlations for CD8+ T cells, NK cells, and MHC-I GSVA score all below -0.05. Abbreviations: BRCA, breast cancer; CESC, cervical squamous cell carcinoma; HNSC, head and neck squamous cell carcinoma; LIHC, liver hepatocellular carcinoma; LUAD, lung adenocarcinoma; LUSC, lung squamous cell carcinoma; MESO, mesothelioma; OV, ovarian cancer; PAAD, pancreatic adenocarcinoma; PCPG, pheochromocytoma and paraganglioma; PRAD, prostate cancer; READ, rectum adenocarcinoma; SARC, sarcoma; SKCM, skin cutaneous melanoma; STAD, stomach adenocarcinoma; TGCT, testicular germ cell tumors; THCA, thyroid carcinoma; UCS, uterine carcinosarcoma.
