# Supplementary material for: Bioinformatic-Experimental Screening Uncovers Multiple Targets for Increase of MHC-I Expression through Activating the Interferon Response in Breast Cancer
Source: Int J Mol Sci. 2024 Sep 30;25(19):10546. doi: 10.3390/ijms251910546 (PMC11476581; doi:10.3390/ijms251910546)
Supplement: Supplementary file 1 [file ijms-25-10546-s001.zip › Supplementary Tables-0927-final.pdf]

Supplementary Table S1: The sequences targeted for gRNA of the genes. Related to the Methods.

gRNA	Sequences
g*NC* (Negative Control)	5’-GTAGTCGGTACGTGACTCGT-3’
g*PIP5K1A* (#1)	5’- GTTAGGCATTACCCACACTG -3’
g*PIP5K1A* (#2)	5’- AAGGCTCAACCTACAAACGG -3’
g*NCKAP1* (#1)	5’- AAGAACTTAGAGCACATGTG -3’
g*NCKAP1* (#2)	5’- ACAGTGTACCCTTAGTGACC -3’
g*CRK* (#1)	5’- CCCGGTGAGGGACTGACGGG -3’
g*CRK* (#2)	5’- CGGGGACTATGTGCTCAGCG -3’
g*CYFIP1* (#1)	5’- CATTGAGATGTCGATGCCCT -3’
g*CYFIP1* (#2)	5’- GCAGATGATCCAGATCCGCG -3’
g*PDGFB* (#1)	5’- CGAGTGCAAGACGCGCACCG -3’
g*PDGFB* (#2)	5’- GCGTCTGGTCAGCGCCGAGG -3’
g*SOCS6* (#1)	5’- TGATGGGTACGCTAAAAAGG -3’
g*SOCS6* (#2)	5’- ACGTGGCTGAAAGTATGCGC -3’
g*SOCS7* (#1)	5’- GATGTGGACATTTCTCAGCG -3’
g*SOCS7* (#2)	5’- CACGGAAACGAGCGACGCGC -3’
g*SOCS4* (#1)	5’- CGATCAGATTTAGCCTTTAG -3’
g*SOCS4* (#2)	5’- TGTGTCATAACCACCGACAA -3’
g*PRKAB2* (#1)	5’- GTGGGTTCATGATCCATCAG -3’
g*PRKAB2* (#2)	5’- CTGTTATCCGCTGGTCTGAA -3’
g*MED4* (#1)	5’- AAATAATTAAGTATGCACAT -3’
g*MED4* (#2)	5’- GTGGTAACAGCACACGAGAG -3’
g*SNAPC3* (#1)	5’- TGTTATATTTCATAAGGTAA -3’
g*SNAPC3* (#2)	5’- TCAAAAACTCACACAACTGA -3’
g*TSR1* (#1)	5’- GGTTATCCCAGATCCAATGG -3’
g*TSR1* (#2)	5’- GCCCCCGCTTGAAACATCGG -3’
g*NOP14* (#1)	5’- CAACAGCAACATGAGCCCCG -3’
g*NOP14* (#2)	5’- CTCTAACAGGATGAAGACGG -3’

g*PAXBP1* (#1)	5’- GAGGCCTCGCGAGAACAAAG -3’
g*PAXBP1* (#2)	5’- CTGATTAATGAACTTGAATC -3’
g*URB1* (#1)	5’- CCGAGTATGTGAGAGGCCTG -3’
g*URB1* (#2)	5’- ACATTGACGGTATGTTCCAT -3’
g*GABPA* (#1)	5’- CGGGGAGAAATTCTCTGGAG -3’
g*GABPA* (#2)	5’- CACCACACTCAACATTTCGG -3’
g*CRCP* (#1)	5’- CCGGCCTGTGACTGCTGTGG -3’
g*CRCP* (#2)	5’- AGGTATTTCAGTTACTAACT -3’
g*ZBTB11* (#1)	5’- TGTCATCAGTAGGATGTATA -3’
g*ZBTB11* (#2)	5’- CTTTGCGAGCACATCTTATT -3’
g*UTP20* (#1)	5’- TCGTGGTGTTACCTCATATT -3’
g*UTP20* (#2)	5’- GTAACTGGATGTGTATACCC -3’
g*POLR1A* (#1)	5’- CGTCAATATTGTGTTTGATA -3’
g*POLR1A* (#2)	5’- CACTGCAAGTAAGTATCAGG -3’
g*SON* (#1)	5’- ACGAGCGCTCTATGATGTCC -3’
g*SON* (#2)	5’- GCCAGTTGTAACAATGTCAG -3’
g*SDAD1* (#1)	5’- TCATCACCTAGTACCCCCAG -3’
g*SDAD1* (#2)	5’- CAAGGTACGTTATTATGACG -3’
g*NUP43* (#1)	5’- TGGTGATGTAATGGATTTAC -3’
g*NUP43* (#2)	5’- ACAGCATGTTGTAGCTACTG -3’
g*GPN3* (#1)	5’- CAAGTCAACATCATGACAAA -3’
g*GPN3* (#2)	5’- GAGCACCTACTGTGCCACCA -3’
g*WDR5* (#1)	5’- CCGTGTCTTTTGTGAAGTTC -3’
g*WDR5* (#2)	5’- TTCAGTTTGACGAAAGCGTG -3’
g*PTCD3* (#1)	5’- CTGTGGACATGTTTGATCAG -3’
g*PTCD3* (#2)	5’- CAGCTGTGCCTTATGTGTTT -3’
g*PTPN11* (#1)	5’- ATGTTATGATTCGCTGTCAG -3’
g*PTPN11* (#2)	5’- TTATAAGAAGAATCCTATGG -3’
g*UTP25* (#1)	5’- TGGTGGTAGCGATGTCAGTG -3’
g*UTP25* (#2)	5’- AGAATATGGATCAGATCCCG -3’

g*ELP1* (#1)	5'- CCGGTCTGTCATTCACCATT -3'
g*ELP1* (#2)	5'- CAGCAATCATGTGTCCCATG -3'
g*NFYB* (#1)	5'- ATATCTTCCAATAGCAAACG -3'
g*NFYB* (#2)	5'- TGCCATACCTCAAACGGGAA -3'
g*DIS3* (#1)	5'- CCGTATATAAACGCATCCGA -3'
g*DIS3* (#2)	5'- GTGTTCCTTAAAATGTGACG -3'
g*MED12* (#1)	5'- TGGTTTGGCACTACTCACTG -3'
g*MED12* (#2)	5'- CGACACGTGCAGTACGCCAC -3'
g*USP39* (#1)	5'- GGGTTTGAAGTCTCACGCCT -3'
g*USP39* (#2)	5'- CATGAGATGCTTCAGGCAGT -3'
g*TBP* (#1)	5'- ACGTCCCAGCAGGCAACACA -3'
g*TBP* (#2)	5'- GATAAGAGAGCCACGAACCA -3'
g*LETM1* (#1)	5'- ATGGATCGACACCAAGATCG -3'
g*LETM1* (#2)	5'- GTCACGGAAGACCGCCTGAG -3'
g*BIRC6* (#1)	5'- CGTAAATCTGAAAACCTCCG -3'
g*BIRC6* (#2)	5'- CATGTAGGCTATAGGTGGGC -3'
g*PTK2* (#1)	5'- CGCAGTCATTTATCATCAGA -3'
g*PTK2* (#2)	5'- ATGTGGGAGATACTGATGCA -3'
g*EXOC1* (#1)	5'- GCATATATTCTAGAACCACA -3'
g*EXOC1* (#2)	5'- TGTTGGATATGGGAAACATG -3'
g*UBQLN4* (#1)	5'- CGATCGAGCCTCGGTCAAGG -3'
g*UBQLN4* (#2)	5'- CTGGCAGTGCCTCTTCAGAT -3'

Supplementary Table S2: Primer Sequences used for RT-qPCR, Related to Methods

Name	Forward Primer (5' to 3')	Reverse Primer (5' to 3')
GAPDH	GACAAGCTTCCCGTTCTCAG	GAGTCAACGGATTTGGTCGT
IFNB1	TGACTATGGTCCAGGCACAG	TTGTTGAGAACCTCCTGGCT
IFIT1	GCCTTGCTGAAGTGTGGAGGAA	ATCCAGGCGATAGGCAGAGATC
RSAD2	CCAGTGCAACTACAAATGCGGC	CGGTCTTGAAGAAATGGCTCTCC
CXCL10	GGTGAGAAGAGATGTCTGAATCC	GTCCATCCTTGGAAGCACTGCA
HLA-A	AGATACACCTGCCATGTGCAGC	GATCACAGCTCCAAGGAGAACC
HLA-B	CTGCTGTGATGTGTAGGAGGAAG	GCTGTGAGAGACACATCAGAGC
HLA-C	GGAGACACAGAAGTACAAGCGC	ACATCCTCTGGAGGGTGTGAGA
HLA-DOB	CCAGATGCTGAGCAGTGGAACA	GGTACACTGTCACCTCTGGTTG
PIP5K1A	ACTTACCAGCCATCGGTCTCTG	ACATCAGGACGACCAAGGTGAAC
NCKAP1	CATCTGGCTACTTCGTCATGCAG	ACAGGTCCGTATTTCCTCACATG
CYFIP1	TACGAGACGCTGCTGAAGCAGA	TCGTCCAATCGCCAGTTCTAGG
DIS3	GTAGACCCACCAGGATGTACTG	CACAGTTGTTCCTCTTCTGGCTG
TBP	TGTATCCACAGTGAATCTTGGTTG	GGTTCGTGGCTCTCTTATCCTC
EXOC1	CCTATGTCTGGCAGAACAGGAC	GCCGTGATAATGTTCCTCCATCC
